# Thermal Stability and Decomposition Mechanism of PLA Nanocomposites with Kraft Lignin and Tannin

**DOI:** 10.3390/polym13162818

**Published:** 2021-08-22

**Authors:** Nina Maria Ainali, Evangelia Tarani, Alexandra Zamboulis, Klementina Pušnik Črešnar, Lidija Fras Zemljič, Konstantinos Chrissafis, Dimitra A. Lambropoulou, Dimitrios N. Bikiaris

**Affiliations:** 1 Laboratory of Chemistry and Technology of Polymers and Dyes, Department of Chemistry, Aristotle University of Thessaloniki, GR54124 Thessaloniki, Greece; nsainali@chem.auth.gr (N.M.A.); azamboulis@gmail.com (A.Z.); 2 Laboratory of Environmental Pollution Control, Department of Chemistry, Aristotle University of Thessaloniki, GR54124 Thessaloniki, Greece; dlambro@chem.auth.gr; 3 Department of Physics, Aristotle University of Thessaloniki, GR54124 Thessaloniki, Greece; etarani@physics.auth.gr (E.T.); hrisafis@physics.auth.gr (K.C.); 4 Faculty of Mechanical Engineering, University of Maribor, 2000 Maribor, Slovenia; klementina.pusnik@um.si (K.P.Č.); lidija.fras@um.si (L.F.Z.)

**Keywords:** poly(lactic acid), nanocomposites, tannin, lignin, thermal degradation kinetics, decomposition mechanism, pyrolysis

## Abstract

Packaging applications cover approximately 40% of the total plastics production, whereas food packaging possesses a high proportion within this context. Due to several environmental concerns, petroleum-based polymers have been shifted to their biobased counterparts. Poly(lactic acid) (PLA) has been proved the most dynamic biobased candidate as a substitute of the conventional polymers. Despite its numerous merits, PLA exhibits some limitations, and thus reinforcing agents are commonly investigated as fillers to ameliorate several characteristics. In the present study, two series of PLA-based nanocomposites filled with biobased kraft-lignin (KL) and tannin (T) in different contents were prepared. A melt–extrusion method was pursued for nanocomposites preparation. The thermal stability of the prepared nanocomposites was examined by Thermogravimetric Analysis, while thermal degradation kinetics was applied to deepen this process. Pyrolysis–Gas Chromatography/Mass Spectrometry was employed to provide more details of the degradation process of PLA filled with the two polyphenolic fillers. It was found that the PLA/lignin nanocomposites show better thermostability than neat PLA, while tannin filler has a small catalytic effect that can reduce the thermal stability of PLA. The calculated Eα value of PLA-T nanocomposite was lower than that of PLA-KL resulting in a substantially higher decomposition rate constant, which accelerate the thermal degradation.

## 1. Introduction

Poly(lactic acid) (PLA), the so-called “polymer of the 21st century”, is one of the most dynamic polymers conforming to a circular and green economy and applied in a wide spectrum of applications due to its exceptional peculiarities [1,2,3]. Among them, its biodegradability, biocompatibility, bioabsorption, versatility and advanced mechanical properties are outpaced, whereas its origin from biomass including corn, sugarcane and other renewable resources should also be remarked upon. One of the most sufficient pathways to synthesize PLA includes the ring opening polymerization of lactide, which is formed by lactic acid; a product of agricultural compounds fermentation [4]. The extended range of its applications can be outlined by its use from the biomedical and bioengineering field [5,6,7] to packaging and everyday-life products [1,8,9,10]. In a more specific context, this aliphatic polyester has been employed for the construction of 3D-printed structures utilized to fulfil several purposes [11,12,13], whereas its application for the preparation of materials with advanced antimicrobial and antioxidant surfaces should also be highlighted [8,14,15,16].

However, since every coin has two sides, PLA downsides are mainly centred on its poor hydrophilicity and toughness, inadequate gas barrier properties, slow degradation rate, low thermal conductivity and weak crystallization [14,17]. Since properties such as mechanical and enzymatic hydrolysis are dependent on the crystallinity of the polymer, it can be concluded that crystallinity of PLA-possessing a commonly semicrystalline nature affects significantly its performance. In fact, polymeric chain architecture greatly affects the ability of nucleation, and thus, the value of crystallinity. Concerning the PLA counterpart, the L-/D- lactide isomers ratio and the macromolecular extent also play a crucial role in the degree of crystallinity [18]. To overcome the limitations attributed to crystallinity and tune the relative properties desired for specific applications, scientific research has been directed towards the synthesis of PLA nanocomposites with several types of nanoscale fillers, including metals, metal oxides, carbon nanotubes and several other clays [10,19,20,21,22,23,24,25,26,27,28]. In general, nano-sized fillers are proved to be excellent agents to boost several properties of polymers, including thermal stability, degradation efficiency, optical, mechanical and permeation properties [29,30,31,32]. In this context, several studies were published concerning the combination of lignin with PLA, since such composite materials exhibit promising characteristics including antioxidant, antifungal and antimicrobial properties; resistance to UV exposure; and fire-retardant capacities [33]. Tannins were also proven to be dynamic polymer additives due to their antioxidant, stabilizing and UV-protective characteristics; nevertheless, tannins were scarcely explored within the context of PLA composites [34]. Nevertheless, some limitations and gaps occur in the field, since the preparation of composite materials with synergistic and advanced properties is a tough aspect.

In detail concerning the relative works, Spiridon et al. [35] and Fereira da Silva et al. [36] have been reported that the addition of lignins into the PLA matrix cause an increase in thermal stability of PLA because of the good adhesion between matrix and filler. However, in both cases, a deterioration in the tensile and impact strength of the composites was demonstrated, especially for fillers loading above 5 wt%, whereas in the second case a decrease in the degree of crystallinity was also observed. Park et al. [37] synthesized PLA-lignin and lignin plasticized composites. They found that the filler decreases the thermal degradation rate against temperature. Zhai et al. [38] demonstrated that the onset of thermal degradation temperature and maximum thermal degradation temperature of PLA composites decrease with increasing filler content. Concerning the PLA-T composites, Anwer et al. [39] and Liao et al. [40] have shown that the onset of thermal degradation of PLA-T composites occurs at a slightly lower temperatures than neat PLA.

Previously, our team reported on the preparation of eight PLA-based nanocomposites with two polyphenolic and natural fillers, namely, kraft-lignin (KL) and tannin (T) at several contents, for the amelioration of their antioxidant character [19]. The detailed preparation, as well as the characterization of the PLA composites with lignin and tannin by several methods, was described previously [19]. Since the results of the aforementioned study revealed the effective addition of the two fillers into the PLA-matrix through the strong suppression of the crystallization time and before the investigation of antioxidant behaviour of the most promising composite materials, the examination of thermal stability, thermal degradation pathway and kinetic parameters has been also considered crucial for the thermal stability control.

The main purpose of the current manuscript was to observe and report thoroughly for the first time the decomposition mechanism of PLA in the presence of KL and T, that could be potential appropriate for packaging applications. To achieve this goal, thermogravimetric analysis (TGA) was initially employed in order to study the effect of the two different polyphenolic fillers on the thermal stability of the PLA matrix and to select the proper temperatures for the pyrolysis tests. Pyrolysis–gas chromatography/mass spectrometry (Py–GC/MS) was also utilized for the analysis of the compounds during the thermal decomposition and the representation of the detailed degradation mechanism of the samples. Additionally, the isoconversional differential method and multivariate non-linear regression method were used in order to determine the effective activation energy and the kinetic triplet (the conversion function f(α), the pre-exponential parameter A and the activation energy) of the degradation reactions. It is noteworthy to mention that the investigation and identification of degradation products are critical for the research of new materials, because they can be formed during treatment processes, and could lead to undesired colouring. A thorough understanding of the degradation mechanism could enhance the selection of thermal stability enhancing agents.

## 2. Materials and Methods

### 2.1. Materials

For the preparation of the nanocomposites, PLA of molar mass Mw~75 kg/mol with ~96% of L- and ~4% of D- forms was employed, and was obtained from Plastika Kritis S.A. (Iraklion, Greece). As reported in our previous study [19], for this molecular weight and based on titration measurements, the fractions of –COOH and –OH groups of PLA were calculated as, approximately, 3 meq/kg and 17 meq/kg, respectively. Tannin (T) and Kraft-Lignin (KL) were purchased from Sigma-Aldrich. According to dynamic light scattering (ZetaSizer 5000, Malvern company, Worcestershire, United Kingdom) measurements, the average particle size of the powders, which were measured in water, was found to be 612 nm for kraft-lignin (polydispersity index, PDI~0.64) and 454 nm for tannin (PDI~0.44).

### 2.2. Preparation of PLA Nanocomposite Materials with Kraft Lignin and Tannin

A melt-mixing via extrusion method was applied for the preparation of the PLA-based nanocomposites filled with tannin and lignin, with a previous overnight step of drying under vacuum, at 110 °C. Dried PLA and tannin or lignin at several wt. fractions were melt mixed in a Haake-Buchler reomixer (model 600) (Haake-Buchler Instruments Ltd., Saddle Brooke, NJ, USA) equipped with roller blades and a mixing head with a volumetric capacity of 69 cm^3^, operating at 195 °C and 30 rpm for 10 min. In total, six nanocomposites were prepared, i.e., with four different lignin contents, 1.0, 2.5, 5.0 and 10.0 wt.%, as well as two different concentrations for tannin nanocomposites, 1.0 and 2.5 wt.%.

### 2.3. Characterization Techniques

#### 2.3.1. Thermogravimetric Analysis (TGA)

Thermogravimetric analysis (TGA) of PLA nanocomposites was performed by a SETARAM SETSYS TG-DTA 16/18 instrument. The samples (6 ± 0.5 mg) were placed in alumina crucibles, while an empty alumina crucible was used as reference. To eliminate the buoyancy effect, a blank measurement was performed and subsequently was subtracted by the experimental curve. For the kinetic analysis study [41], PLA nanocomposites were heated from 25 °C to 600 °C, in a 50 mL/min flow of N_2_ at heating rates of 5, 10, 15 and 20 °C/min. Continuous recordings of sample temperature, sample mass, its first derivative and heat flow were taken. Thermal degradation kinetic analysis of the PLA nanocomposites was performed using NETZSCH Kinetics Neo software (NETZSCH, Selb, Germany). This model-fitting kinetic approach aims to find a kinetic model with minimal adjustable parameters which quantitatively describes the kinetics of the complete degradation reaction.

#### 2.3.2. Pyrolysis-Gas Chromatography/Mass Spectrometry (Py-GC/MS) Study

For Py-GC/MS analysis of the PLA nanocomposites, a very small amount of each material was “dropped” initially into the “Double-Shot” EGA/PY 3030D Pyrolyzer (Frontier Laboratories Ltd., Fukushima, Japan) using a CGS-1050Ex carrier gas selector. For pyrolysis analysis (flash pyrolysis), each sample was placed into the sample cup which afterwards fell into the Pyrolyzer furnace. The pre-selected pyrolysis temperature was differentiated for each nanocomposite sample, while the GC oven temperature was programmed at 50 °C for 2 min, followed by a stepped increase to 200 °C with a heating rate of 5 °C/min, where it was held for 8 min, and then the temperature was increased at 300 °C by a rate 20 °C/min, where it was held for 5 min. Sample vapours generated in the furnace were split (at a ratio of 1/50), a portion moved to the column at a flow rate of 1 mL/min, pressure 53.6 kPa and the remaining portion exited the system via the vent. The pyrolyzates were separated using temperature programmed capillary column of a Shimadzu QP-2010 Ultra Plus (Kyoto, Japan) gas chromatogram and analysed by the mass spectrometer MS-QP2010SE of Shimadzu (Kyoto, Japan). Ultra-ALLOY^®^ metal capillary column from Frontier Laboratories LTD (Fukushima, Japan) was used containing 5% diphenyl and 95% dimethylpolysiloxane stationary phase, column length 30 m and column ID 0.25 mm. For the mass spectrometer, the following conditions were used: ion source heater 200 °C, interface temperature 300 °C, vacuum 10^−4^–10^0^ Pa, m/z range 45–500 amu (atomic mass unit) and scan speed 10,000. The ion gas chromatograms and spectra retrieved by each experiment were subjected to further interpretation through Shimadzu and Frontier post-run software. The chromatogram and spectra retrieved by each experiment were subject to further interpretation through Shimadzu (NIST11.0) and Frontier (F-Search software 4.3) post-run software. Identification was recognized depending on the similarity percentage (minimum value of 80%) between average mass spectra on the entire chromatogram.

## 3. Results and Discussion

### 3.1. Thermogravimetric Analysis (TGA)

**Figure 1:**
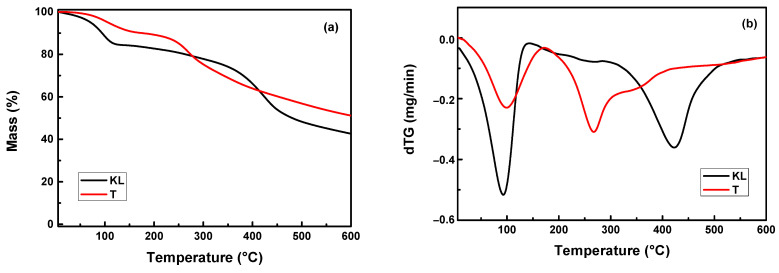
(**a**) TGA thermograms and (**b**) dTG curves of KL and T samples at a heating rate of 20 °C/min under a nitrogen atmosphere.

The thermal degradation of KL, T and PLA nanocomposites filled with the two different polyphenolic fillers was studied by TGA analysis. Figure 1 shows the TGA thermograms and derivative mass loss (dTG) curves of KL and T. The temperatures corresponding to 2.5% and 5% mass loss were found to be 49.2 °C and 71 °C for KL, as well as 83.1 °C and 106.1 °C for T sample. KL shows a small weight loss of ~12% below 100 °C due to water evaporation and the main degradation step between 200 °C and 500 °C because of the gradual degradation, and emission of phenol molecules [36,42]. Tannin exhibits a three-step degradation process that includes the loss of adsorbed water, the decomposition of linkage between flavonoid units and pyrolytic degradation [34]. Additionally, the residual mass of KL and T fillers reaches ~41% and 50% at 600 °C, indicating that the fillers do not fully degrade at that specific temperature. Based on these thermograms, it is clear that lignin is more stable than tannin. The maximum rate of the main decomposition steep of lignin is recorded at 425 °C while in tannin at 260 °C (Figure 1b).

**Figure 2:**
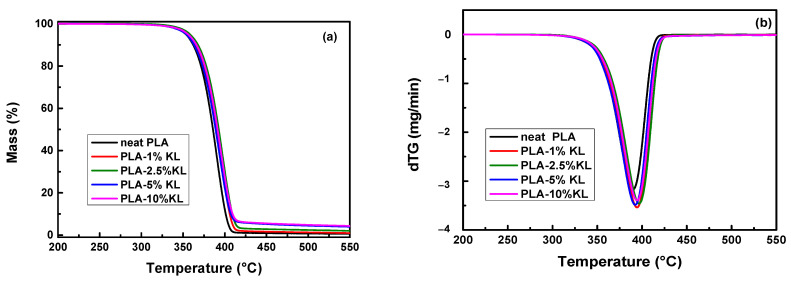

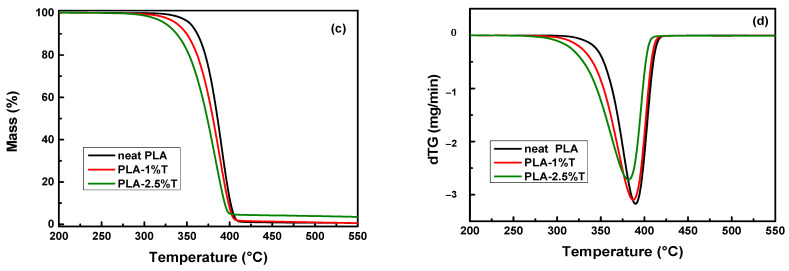
TGA thermograms and dTG curves of PLA nanocomposites filled with (**a**,**b**) KL and (**c**,**d**) T at a heating rate of 20 °C/min under a nitrogen atmosphere.

Figure 2 presents the TGA thermograms and dTG curves of PLA nanocomposites filled with the two different polyphenolic fillers at a heating rate of 20 °C/min, while the TGA results of all the studied nanocomposites are shown in Table 1. It can be seen from the TGA curves, Figure 2a,b, that PLA/KL nanocomposites show good thermostability since no significant mass loss (<0.5%) occurs until 310 °C. It should be mentioned that the mass loss curves of the PLA nanocomposites appear to be almost identical with a one-step procedure yielding the same curve. Additionally, the residue content of the PLA nanocomposites was found to be close to the added filler content due to the fact that the fillers do not fully decompose until 600 °C (Figure 1). The temperatures corresponding to 0.5%, 2.5% and 5% mass loss, as well as the char residue of the samples at 600 °C, are shown in Table 1. An increase in thermal stability of PLA-lignin nanocomposites was found due to the presence of aromatic phenyl groups and hydroxyl groups [35,42,43,44]. However, Zhai et al. and Park et al. [37,38] found that both the onset thermal degradation temperature and the maximum thermal degradation temperature of PLA nanocomposites decrease with increasing filler content because of the lower onset thermal degradation temperature and maximum thermal degradation temperature of lignin compared to neat PLA. From the dTG curves, the highest decomposition rate of neat PLA-1%KL, PLA-2.5%KL, PLA-5%KL and PLA-10%KL nanocomposites was found to be 394.4 °C, 396.7 °C, 392.2 °C and 394.4 °C, respectively. Additionally, the PLA-1%T and PLA-2.5% nanocomposites decompose at 283 °C and 266 °C, respectively, with the highest decomposition rate at 387.4 °C and 382.2 °C, which are significantly lower than the previous ones. So, T filler has a strong catalytic effect and can markedly reduce PLA thermal stability because of the presence of highly polarized hydroxyl groups and low polarity PLA matrix, resulting in poor interfacial adhesion and dispersion of T in the PLA matrix [19,34,40]. Concerning the char residue of the nanocomposite materials, it was shown that for all the samples, the addition of both the two polyphenolic fillers tended to rise the char residue, as reported by previous studies [42,45].

As a final point, the difference between the behaviour of KL and T regarding the thermal stability of the PLA composites may be ascribed to their slightly differentiated structures. In fact, T is a smaller compound with a lower molecular weight than KL, while it contains on its structure fewer aromatic groups and higher amount of phenolic -OH groups in contrast to the methoxy- groups of KL. Due to these differences, tannin decomposes at lower temperatures than KL (Figure 1) and this could also affect the thermal stability of PLA/T nanocomposites.

### 3.2. Study of the Degradation Mechanism with Py-GC/MS

Analytical techniques based on pyrolysis have proven particularly successful to investigate the degradation process of several polymers as well as their blends, since information is provided at a molecular level on these complex polymeric systems by examining their pyrolytic products. In fact, the latter are small volatile molecules formed by selective bond cleavage induced by thermal energy [46]. Once coupled with gas chromatography (GC) and mass spectrometry (MS), thermoanalytical pathway is highly sensitive, requires small masses and a minimum sample preparation while the analysis time is generally short.

**Figure 3:**
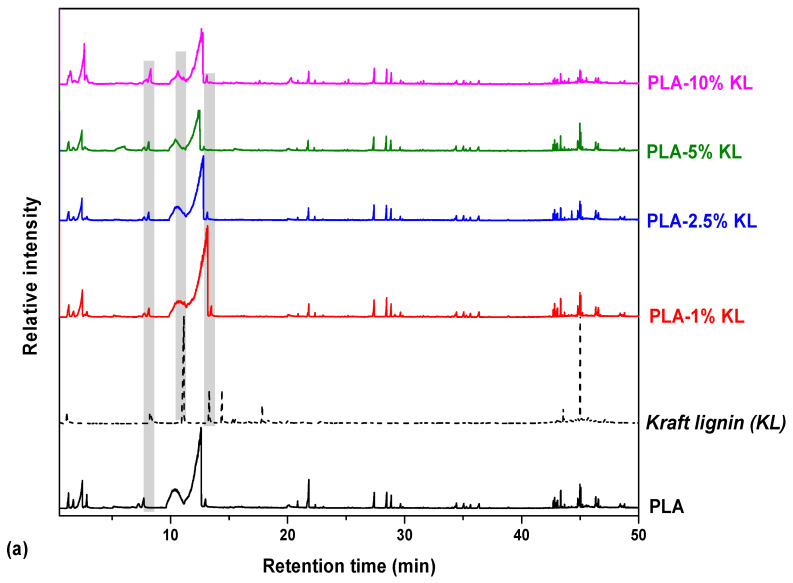

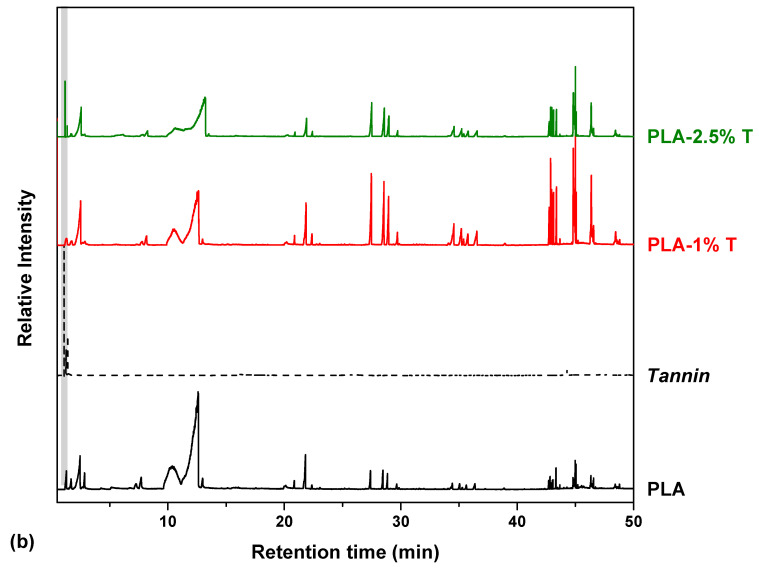
Total ion gas chromatograms of (**a**) neat PLA, KL, PLA nanocomposites with 1, 2.5, 5 and 10% wt. KL content and (**b**) neat PLA, T and PLA nanocomposites with 1 and 2.5% wt. T.

**Figure 4:**
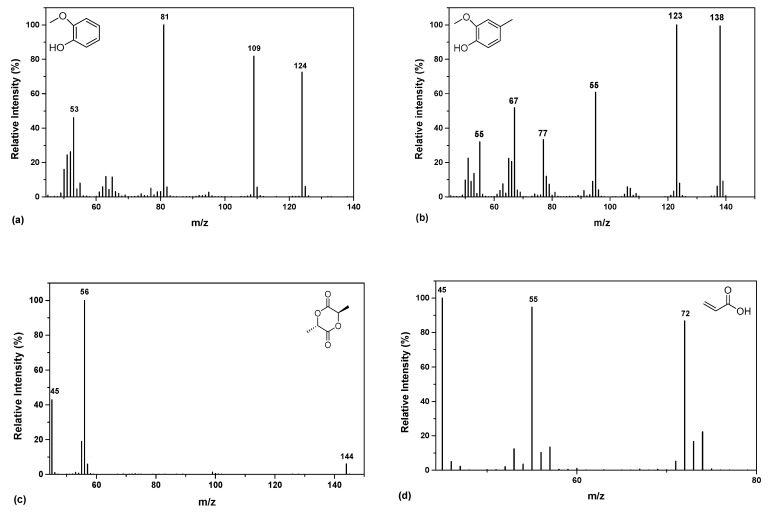
Mass spectra of (**a**) 2-methoxy-phenol, (**b**) 2-methoxy-4-methylphenol, (**c**) d,l-lactide and (**d**) 2-propenoic acid.

Herein, in order to investigate the degradation process of the prepared nanocomposites based on a PLA matrix and the two polyphenolic fillers, namely kraft lignin and tannin, temperatures related to each sample’s degradation peak were chosen for Py-GC/MS analysis. This thermoanalytical approach gives an insight into the optimal parameters required for the thermal stability control, and thus, is a useful tool for the preparation of the PLA nanocomposites for further packaging applications. Moreover, in an attempt to investigate how the pyrolytic profile of PLA materials is affected by the incorporation of the two fillers in different contents is also illustrated. All nanocomposites were pyrolyzed at a temperature range of 387–397 °C (Table 2), while the recorded chromatographs of the degradation products are shown in Figure 3. The most characteristic pyrolysis products were identified through their MS spectra and are presented in Table 2, while some indicative mass spectra of the pyrolytic compounds of KL and PLA-composites are depicted in Figure 4.

Generally, thermal degradation of thermoplastic polyesters takes place via heterolytic and homolytic scission of their aliphatic fragments. At small retention times (Rt), more volatile compounds with low molecular weight are found, whereas as retention time increases, larger and more complex compounds are identified, such as dimer, trimer and tetramer structures. The foremost pyrolyzates correspond to characteristic products of β-scission reactions including compounds with vinyl- and carboxyl- end groups. Regarding PLA, the complexity of its thermal decomposition has been extensively explored in literature [47,48,49,50,51,52,53,54]. In brief, at temperatures above 200 °C, intramolecular trans-esterification processes hold an outpacing position with the formation of lactide and cyclic oligomers due to cis-elimination proceeds, while the formation of acrylic acid and other oligomers is due to the β-scission reactions. Further fragmentation leads to the final formation of acetaldehyde and CO_2_ [47].

The principal thermal degradation products of PLA are depicted in Table 2. Specifically, according to the chromatogram of PLA (Figure 3), the first identified peak corresponds to acetaldehyde, while 2-propenoic acid (Figure 4d) and 2,3-pentanedione can also be detected due to the β-scission mechanism. Figure 3 exhibits the presence of two peaks (Rt = 10.25 and 12.47 min), assigned to the fragments with m/z of 45, 56 and 144 (Figure 4c). The aforementioned peaks conformed to meso-lactide and d,l-lactide structures, respectively, whereas peaks appearing sporadically at retention times higher than 20 min were ascribed to larger cyclic lactide oligomers [52,53].

Among the products of KL, phenol derivatives and aromatic hydrocarbons were identified, with the most characteristic peak the relative found at Rt = 11.15 min, attributed to 2-methoxy-4-methylphenol (Figure 4b) [55,56,57]. Concerning the chromatographs of Figure 3a, all the PLA nanocomposites released compounds of low molecular weight at low retention times, including acetaldehyde, 2-propenoic acid and 2,3-pentanedione. In retention times within the range 10.25–12.47 min, the two distinctive peaks of PLA are depicted; meso-lactide and d,l-lactide. A noteworthy remark about the PLA nanocomposites is that as the content of KL increases the morphology of the peaks is slightly modified, whereas the meso-lactide intensity increases from 22.5% (1% KL) to 30% (10% KL) in comparison with the relative of d,l-lactide counterpart. Moreover, a new small peak assigned to 2-methoxy-4-methylphenol is displayed in Figure 3 at Rt = 11.18 min, mainly due to the larger content of the filler in this material. Generally, for the PLA nanocomposites with KL as well as T, it could be noticed that their pyrolysis products identified were identical, presenting that KL and T as fillers do not affect the degradation products of PLA formed at low content. In detail, both fillers possess aromatic structure, with KL containing mainly hydroxy- and methoxy- groups, and T holding plentiful hydroxyl groups (phenolic) on its architecture. From the other hand, PLA has a poor hydrophilic structure which may prevent the interactions between the polymer matrix and the two fillers. Due to this aforementioned fact, the addition of both phenolic compounds seems that do not affect the thermal degradation mechanism of PLA.

### 3.3. Kinetic Analysis Based on Thermogravimetric Data

The degradation mechanism of PLA-1% nanocomposites filled with the two different polyphenolic fillers was described by calculating the degree of conversion (α) and kinetic parameters. For this reason, the isoconversional methods and the model fitting methods were used. The mass curves were recorded at various heating rates (5, 10, 15 and 20 °C/min) under nitrogen. The reaction rate can be defined using the general equation of solid-state reactions [41]:

(1) $$\frac{d\alpha }{\mathrm{dt}}=k\left(T\right)f\left(\alpha \right)$$

where k(T) is the reaction rate constant, f(α) is the reaction model and α is the degree of conversion. The term degree of conversion a refers to the ratio of the actual mass loss at a given temperature Δm to the total mass loss Δm_tot_ which occurs after the completion of the degradation process [22]:

(2) $$\alpha =\frac{m_{0}-m}{m_{0}-m_{f}}=\frac{\Delta m}{{\Delta m}_{\mathrm{tot}}}$$

The kinetic model, f(α) is an algebraic expression that describes the kinetics of the solid-state reaction. The dependence of temperature on the rate of reaction is defined by the Arrhenius equation, $k\left(T\right)={\mathrm{Ae}}^{-E/\mathrm{RT}}$, where E is the apparent activation energy (kJ/mol), R the gas constant (8.314 J/mol·K), A the pre-exponential factor (s^−1^) and T the absolute temperature (K).

The isoconversional methods are divided into differential and integral methods. The most frequently used methods are the differential isoconversional method of Friedman [58], the integral isoconversional method of Vyazovkin [59], and the integral isoconversional method of Ozawa, Flynn and Wall (OFW) [60].

The differential isoconversional Friedman’s method can be described [58]:

(3) $$\ln\left[\beta_{i}{\left(\frac{d\alpha }{\mathrm{dt}}\right)}_{\alpha ,i}\right]=\ln\left[f\left(\alpha \right)A_{\alpha }\right]-\frac{E_{\alpha }}{{\mathrm{RT}}_{\alpha ,i}}$$

where A is the pre-exponential factor and β is the heating rate. To obtain the activation energy E_α_ values at a constant conversion function, the slope of the straight lines of the plot ln[βi(dα/dt)a i] vs. 1/T_α,i_ should be calculated. Differential methods have the advantage of requiring no approximations and applying them to any temperature program. However, a determination limit on the baseline causes limited accuracy, and they occasionally display numerical instability compared to integral ones.

An isoconversional nonlinear method has been proposed by Vyazovkin in order to calculate the Eα [59]:

(4) $$\Phi \left({Ε}_{\alpha }\right)=\overset{n}{\underset{i=1}{\sum }}\overset{n}{\underset{j\neq 1}{\sum }}\frac{J\left[E_{\alpha },{Τ}_{i}\left(t_{\alpha }\right)\right]}{J\left[E_{\alpha },{Τ}_{j}\left(t_{\alpha }\right)\right]}\;$$

where the indexes i and j denote set of experiments performed under different heating rates, n is the total number of experiments and J is evaluated over small intervals of Eα variation:

(5) $$J\left[E_{\alpha },{Τ}_{i}\left(t_{\alpha }\right)\right]=\overset{t_{\alpha }}{\underset{t_{\alpha -\Delta \alpha }}{\int }}\exp\left[\frac{-E_{\alpha }}{{\mathrm{RT}}_{i}\left(t\right)}\right]\mathrm{dt}$$

Τhe value that minimizes the function Φ(Eα), Equation (4), is used for the calculation of Eα. The time tα,i and temperature T_α,i_ of selected α values are found by an exact interpolation utilizing a Lagrangian algorithm for each i-th temperature program.

The Ozawa, Flynn and Wall equation can be presented as [61,62,63]:

(6) $$\ln\left(\beta_{i}\right)=\mathrm{Const}-1.052\left(\frac{E_{\alpha }}{{\mathrm{RT}}_{\alpha }}\right)$$

where β=dT/dt=const is the heating rate. The index i express the different heating rates that were applied to the experimental data. The activation energy E_α_ values can be obtained by the slope of the ln(βi) vs. 1/T_α_ plots.

**Figure 5:**
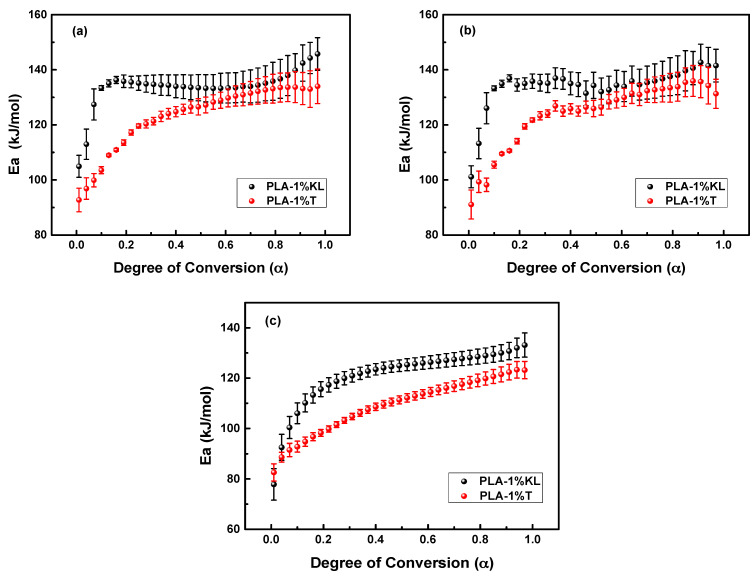
The dependence of activation energy (E_α_) on the extent of conversion (α) for the thermal degradation of PLA-1% nanocomposites filled with KL and T as calculated by (**a**) Friedman method, (**b**) Vyazovkin analysis and (**c**) OFW analysis.

Three iso-conversional methods, the differential isoconversional method of Friedman, the integral isoconversional method of Vyazovkin, and the integral isoconversional method of OFW were used for the calculation of the activation energies [58,59,64,65,66]. Figure 5 shows the E_α_ values of PLA nanocomposites filled with the two different polyphenolic fillers versus the degree of conversion α by using the above-mentioned iso-conversional methods. Τhe E_α_ mean value obtained by using the Vyazovkin method from the TGA curves was found to be in excellent agreement with the values computed by the Friedman method, while the differences in E_α_ values between the OFW and Friedman methods can be explained by a systematic error owing to poor integration [67]. In all cases, the E_α_-dependency suggests that the thermal degradation mechanism is complex, and the degradation kinetics is regulated by different processes at the initial and final stages. The E_α_ values of PLA-1%KL and PLA-1%T nanocomposites present a rapid increase in the first region, while it remains almost constant for greater values. According to the Friedman method, Vyazovkin analysis, and OFW method, the E_α_ mean values of PLA-1%KL nanocomposite for α < 0.1 were found to be 115.2, 114.5, and 103.7 kJ/mol, respectively, while the values were 135.8, 136.4, and 126.0 kJ/mol for α > 0.1. Additionally, the E_α_ values of PLA-1%T nanocomposite (α < 0.3) were 109.3, 109.9 and 96.1 kJ/mol for Friedman method, Vyazovkin analysis, and OFW method, respectively, and 129.6, 130.5, and 115.6 kJ/mol for α > 0.3. The PLA-1%T nanocomposite has lower activation energy than PLA-1%KL, requiring less activation energy to undergo thermal degradation.

**Figure 6:**
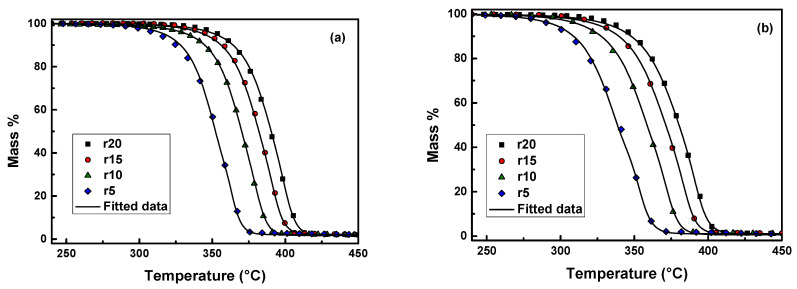
Mass (%) curves of (**a**) neat PLA-1%KL and (**b**) PLA-1%T at heating rates of 5, 10, 15 and 20 °C/min in a nitrogen atmosphere (symbols) and the corresponding fitting curves with the combination of Cn-Cn reaction models (continuous black lines).

The degradation mechanism and the kinetic triplet (E, A, f(α)) of each reaction were determined by using multivariate non-linear regression method or model-fitting method through the comparison of the experimental (four heating rates) and theoretical data. Various reaction models (16 models) were adopted for the fitting to the experimental data at different heating rates. First, a single-step reaction mechanism is assumed to occur, which corresponds to the major mass loss. If the fitting to the experimental data does not yield satisfactory results, two or more mechanisms are combined. In our previous work [14], it was found that the form of the conversion function, which describes well the neat PLA is the mechanism of autocatalysis n-order (Cn):

(7) $$f\left(\alpha \right)={\left(1-\alpha \right)}^{n}\left(1+K_{\mathrm{cat}}X\right)$$

where K_cat_ is the autocatalysis rate constant and *X* is the extent of conversion of the autocatalytic reactions. According to autocatalytic reaction, the thermally induced polymer fragments (reaction products) attack the unreacted parts of the polymer breaking the chemical bonds and causing acceleration of the degradation process. Figure 5 shows that the combination of two consecutive Cn-Cn reaction models fits well the experimental data of PLA-1%KL and PLA-1%T nanocomposites. The calculated parameters (activation energy, pre-exponential factor, and the reaction order) obtained by the tested models are summarized in Table 3. The E_α_ values calculated by the two-step mechanism model were found to be in good agreement with those estimated by the isoconversional methods (Figure 6). The calculated values suggest that the Τ filler, added to the PLA matrix, has led to lower E_α_ values, and resulted in a substantially higher decomposition rate constant. Additionally, the pre-exponential factor A of the PLA-1%T nanocomposite presents lower values in both stages than those of PLA-1%KL following the calculated E_α_ values. This means that the rate constant of PLA-1%T nanocomposite is significantly larger than that of PLA-1%KL, accelerating the thermal degradation. The values of correlation coefficient (R^2^) were found to be 0.99993 and 0.99992 for PLA-1%KL and PLA-1%T nanocomposites, respectively.

## 4. Conclusions

The effect of two natural polyphenolic fillers, namely kraft lignin and tannin on the thermal stability and degradation of PLA was thoroughly evaluated throughout this manuscript. For this reason, TGA in conjunction with Py-GC/MS was effectively employed to investigate the thermal stability, decomposition kinetic and mechanism of PLA nanocomposites. The isoconversional differential method and multivariate non-linear regression method were used for the determination of the effective activation energy and the kinetic triplet of the degradation reactions of PLA nanocomposites. TGA thermographs depicted that PLA/KL nanocomposites present good thermostability, since no significant mass loss takes place until 310 °C. This fact may be attributed to the additional aromatic groups of KL. On the other hand, T filler displayed a severe catalytic effect on the PLA/T nanocomposites and provoked a reduction of PLA thermal stability. Concerning the investigation of the thermal degradation pathway through Py-GC/MS analysis, it was found that for both PLA and its composites with KL and T, intramolecular *trans*-esterification processes hold a principal role during decomposition with the formation of mainly lactide and cyclic oligomers, while cis-elimination provokes the formation of acrylic acid and oligomers. It seems that the addition of both the fillers does not greatly affect the thermal degradation pathway of PLA. Additionally, a two-step mechanism was detected for the thermal degradation of PLA-1%KL and PLA-1%T nanocomposites, which was best fitted by the Cn–Cn model (n-th order model). It was found that the T filler, added in the polymer matrix, decreased the E_α_ values, leading to higher decomposition rate constant compared to that of PLA-KL nanocomposite and accelerating the thermal degradation process.

## Figures and Tables

**Table 1:** TGA results of all studied samples.

Sample	T_0.5_ (°C)	T_2.5_ (°C)	T_5_ (°C)	T_d,max_ (°C)	CR_600_ (%)
Neat PLA	320.9	343.4	353.3	389.7	0.4
PLA-1%KL	320.2	344.1	354.4	394.4	0.6
PLA-2.5%KL	324.6	348.6	358.1	396.7	1.6
PLA-5%KL	310.7	342.6	353.7	392.2	3.4
PLA-10%KL	306.6	345.4	355.9	394.4	3.9
PLA-1%T	290.6	325.7	337.6	387.4	0.4
PLA-2.5%T	282.2	311.2	324.2	382.2	3.1

**Table 2:** Main pyrolysis products evolved during thermal decomposition of PLA, KL (KL), T (T) and their nanocomposites, at different temperatures as identified with mass spectroscopy based on the Py-GC chromatograms.

PLA	Kraft Lignin (KL)	PLA-1%KL	PLA-2.5%KL	PLA-5%KL	PLA-10%KL	Tannin (T)	PLA-1%T	PLA-2.5%T	Mw (amu)	Possible Product
Pyrolysis Temperature										
390 °C	400 °C	394 °C	397 °C	392 °C	394 °C	400 °C	387 °C	382 °C		
Rt (min)										
-	1.16	-	-	-	-	-	-	-	64	Sulphur dioxide
-	-	-	-	-	-	1.19	1.21	1.21	58	Acetone 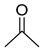
1.27	-	1.26	1.27	1.24	1.23	-	1.29	1.33	44	Acetaldehyde 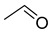
2.45	-	2.46	2.43	2.39	2.64	-	2.49	2.54	72	2-propenoic acid (acrylic acid) 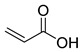
2.83	-	2.87	2.80	2.75	2.79	-	2.71	2.84	100	2,3-pentanedione
-	8.24	-	-	-	8.26	-	-	-	124	2-methoxy-phenol 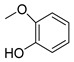
-	11.10	-	-	11.10	11.11	-	-	-	138	2-methoxy-4-methylphenol (creosol) 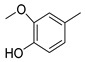
10.25 12.47	-	10.73 13.11	10.63 12.71	10.52 12.77	10.63 12.71	-	10.52 12.61	10.67 13.21	144	meso-lactide or d,l-lactide 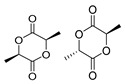
-	13.30	-	-	-	-	-	-	-	164	2-methoxy-3-(2-propenyl)-phenol 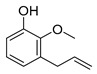
-	14.38	-	-	-	-	-	-	-	180	4-((1E)-3-Hydroxy-1-propenyl)-2-methoxyphenol 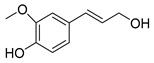
21.74	-	21.82	21.78	21.70	21.80	-	21.90	21.90	202	PLA trimer

**Table 3:** Activation energy, pre-exponential factor and reaction order of PLA nanocomposites.

Sample	Model	Activation Energy, E/kJmol^−1^	Pre-Exponential Factor, logA_1_/s^−1^	Reaction Order/n	Log K_cat_	R^2^
PLA-1%KL	Cn	119.02	5.182	0.694	1.081	0.99993
	Cn	134.15	6.277	1.241	1.381	
PLA-1%T	Cn	102.69	4.086	0.635	0.930	0.99992
	Cn	132.71	6.106	1.210	1.324	

## Data Availability

The data presented in this study are available on request from the corresponding author.
